# Nitric oxide regulates adhesiveness, invasiveness, and migration of anoikis-resistant endothelial cells

**DOI:** 10.1590/1414-431X2021e11612

**Published:** 2022-02-04

**Authors:** A.P.S. Mesquita, M. Matsuoka, S.A. Lopes, P.C.A. Pernambuco, A.S. Cruz, H.B. Nader, C.C. Lopes

**Affiliations:** 1 Departamento de Bioquímica, Universidade Federal de São Paulo, São Paulo, SP, Brasil; 2 Departamento de Ciências Biológicas, Universidade Federal de São Paulo, Diadema, SP, Brasil

**Keywords:** Anoikis resistance, Nitric oxide, Endothelial nitric oxide synthase (eNOS), Metalloproteinase-2 (MMP-2), Endothelial cell

## Abstract

Anoikis is a type of apoptosis that occurs in response to the loss of adhesion to the extracellular matrix (ECM). Anoikis resistance is a critical mechanism in cancer and contributes to tumor metastasis. Nitric oxide (NO) is frequently upregulated in the tumor area and is considered an important player in cancer metastasis. The aim of this study was to evaluate the effect of NO on adhesiveness, invasiveness, and migration of anoikis-resistant endothelial cells. Here, we report that anoikis-resistant endothelial cells overexpress endothelial nitric oxide synthase. The inhibition of NO release in anoikis-resistant endothelial cells was able to decrease adhesiveness to fibronectin, laminin, and collagen IV. This was accompanied by an increase in cell invasiveness and migration. Furthermore, anoikis-resistant cell lines displayed a decrease in fibronectin and collagen IV protein expression after L-NAME treatment. These alterations in adhesiveness and invasiveness were the consequence of MMP-2 up-regulation observed after NO release inhibition. The decrease in NO levels was able to down-regulate the activating transcription factor 3 (ATF3) protein expression. ATF3 represses *MMP-2* gene expression by antagonizing p53-dependent trans-activation of the MMP-2 promoter. We speculate that the increased release of NO by anoikis-resistant endothelial cells acted as a response to restrict the MMP-2 action, interfering in *MMP-2* gene expression via ATF3 regulation. The up-regulation of nitric oxide by anoikis-resistant endothelial cells is an important response to restrict tumorigenic behavior. Without this mechanism, invasiveness and migration potential would be even higher, as shown after L-NAME treatment.

## Introduction

The dynamic balance between cell proliferation, differentiation, and apoptosis is very important for the maintenance of cell and tissue homeostasis (1). Adherent cells depend on continuous signals mediated by interactions with other cells or ECM proteins to survive. Several cellular functions are regulated by the transduction of signals originating from extracellular interactions, and loss of contact induces a specific form of apoptosis, anoikis (2,3). Anoikis activation is an important physiological mechanism that ensures normal development and homeostasis of tissues by preventing detached cells from reattaching and colonizing an inappropriate matrix, thus acting as a protective tool against dysplastic cell growth. Dysregulation of anoikis, such as the acquisition of anoikis resistance, is a critical step in oncogenic epithelial-mesenchymal transition and contributes significantly to tumorigenesis and especially to tumor metastasis (3). Tumor cells that acquire the ability to resist anoikis can survive after detaching from their original matrix and invade the blood or lymphatic circulation, promoting their metastatic spread to distant sites (3,4).

The acquisition of anoikis resistance by endothelial cells leads to alterations in cell morphology, increase in cell proliferation rate, poor adhesion to extracellular matrix (ECM) molecules such as fibronectin, laminin, and collagen IV, and dysregulation of the cell cycle. Furthermore, anoikis-resistant endothelial cell lines display higher invasiveness, lower rate of apoptosis, and overexpression of molecules involved in the PI3K/Akt and Ras/ERK cell signaling pathways compared to the parental cell line, endothelial cell (EC). This is accompanied by extensive ECM remodeling and alterations in heparan sulfate and chondroitin sulfate levels, as well as changes in the syndecan-4 and heparanase expression (2,5). The above alterations make endothelial cells resistant to anoikis and behave similar to tumorigenic endothelial cells (EJ-ras EC). Tumorigenic cells adapt their metabolism in order to produce several molecules and promote cell proliferation, maintenance, and activation of the metastatic cascade. Studies on non-malignant and malignant cell lines suggest that several molecules associated with cell signaling, such as nitric oxide (NO), are altered in metastatic cells (6).

NO is a free radical gas that is considered an important signaling molecule in several of physiological processes. The NO cellular production from L-arginine occurs in the cytoplasm. The endothelial enzyme NOS (eNOS or NOS3) is constitutively expressed by vascular endothelial cells (7). Physiologically, NO, which is synthetized by eNOS, acts as a regulator of vascular tone and an inhibitor of platelet adhesion (8).

Several studies demonstrate that NO is involved in the pathophysiology of some diseases such as inflammatory processes and diabetes and is considered to be an important player in cancer and metastasis (7). NO can modulate several vascular characteristics in tumor microcirculation, such as angiogenesis, hemodynamics of established tumor vessels, and vascular permeability (9). NOS expression is positively associated with tumor microvessel density and angiogenic ability in rabbit corneal xenografts (10). Moreover, NO is frequently up-regulated in the tumor area and can also modulate the metastatic capacity of tumor cells (11). Much evidence suggests a stimulatory role of NO in angiogenesis under various conditions including tumor growth. NO is the final mediator of angiogenesis stimulated by vascular endothelial growth factor (VEGF), and it is absolutely required for endothelial cell migration induced by this growth factor (12). However, it is not simple to define the role of NO in metastasis due its dichotomy of effects. NO can produce widely different outcomes in cancer and depends on the concentration range and cell type involved (6).

The metastatic process occurs as a multistage mechanism, beginning with the loss of cell-cell junctions and the release of anoikis-resistant single cells from the tumor, ECM degradation and cell migration, entry into blood or lymphatic vessels, and finally invasion and growth at a secondary site (13). ECM degradation and cell migration are promoted by the action of matrix metalloproteinases (MMPs). MMPs are the main enzymes involved in ECM deg-radation. Their activity is low in normal conditions but increases during repair or remodeling processes and in metastases or inflamed tissue (14).

Matrix metalloproteinase 2 (MMP-2) is implicated in numerous pathogenic processes, including tumor invasion and metastasis (15). Alterations in MMPs expression and activity may be regulated by NO levels. The 72-kDa MMP-2 zymogen can be directly activated by the peroxynitrite (ONOO-) resulting from the pro-oxidant reaction of NO. This activation occurs via a non-proteolytic process involving the S-glutathiolation of a cysteine sulfhydryl fraction in its propeptide (16). Conversely, nitric oxide can inhibit MMP-2 expression via the induction of activating transcription factor 3 (ATF-3) (17).

Here, we report that acquisition of anoikis resistance causes endothelial cells to overexpress eNOS and, consequently, these cells present a high release of NO. Inhibiting NO release could affect the metastatic characteristics of these cells such as invasiveness, adhesiveness, migration, and matrix remodeling. Thus, this work aimed to clarify the role of NO in the tumorigenic behavior of endothelial cells resistant to anoikis.

## Material and Methods

### Cell culture

The endothelial cell lines derived from rabbit aorta (EC), EC transfected with EJ-*ras* oncogene (EJ-*ras* EC), and ECs resistant to anoikis (Adh1-EC and Adh2-EC) were maintained in F-12 medium (Sigma-Aldrich, cat# N6760, USA) supplemented with 10% fetal calf serum (FCS) (Vitrocell, Brazil) at 37°C and 2.5% CO_2_. In order to inhibit NO production, the cells were treated with L-N^G^-nitroarginine methyl ester (L-NAME, Cayman, cat# 80210, USA), an inhibitor of the enzyme eNOS, at a concentration of 2 mM for 1 h.

### Detection and measurement of NO production

An equal number of cells (5×10^5^) were cultured in 60-mm dishes for 24 h. Culture medium was collected for measurements of NO levels. A NO analyzer (NOA 280i, Sievers Instruments, USA) was used for determination of endogenous NO levels. Aliquots of culture medium were injected into a nitrogen-purged chamber containing a reducing agent (1% of NaI in acetic acid), which converts the NO oxidizing product nitrate back to NO. Steady-state micromolar concentrations of NO were calculated from the peak areas of absolute NO detected and compared with a sodium nitrite standard curve as reference. Levels of NO detected in the reaction medium were corrected for background noise by subtracting the amount of NO present in F-12 supplemented with 10% FBS. Experiments were performed in duplicate.

To investigate the role of high NO levels in anoikis-resistant endothelial cells lines we used L-NAME. This L-arginine analogue has been widely applied for several decades in both basic and clinical research as an antagonist of nitric oxide synthase enzymes (NOS) (18).

To evaluate the inhibitory effect of L-NAME in NO production, an equal number (5×10^5^) of EC, EJ-ras EC, Adh1-EC, and Adh2-EC cells were placed on 60-mm plates. After 24 h, the culture medium was replaced with F-12 medium containing L-NAME (2 mM) or only F-12 medium for control samples, for 1 h. The culture medium was collected and levels of NO_2_^-^/NO_3_^-^ were determined by the Griess reaction using a commercial kit from Cayman Chemical Co. (cat# 780001, USA). The azo dye formed as a product of Griess diazotization reaction was spectrophotometrically quantitated based on its absorbance at 570 nm, using the microplate reader EZ Read 400 (Biochrom, UK). The released nitrite values were calculated based on a NaNO_2_ standard curve. All measurements were performed in triplicate.

### Quantitative real-time PCR (qPCR)

For qPCR, 2 μg of total RNA per sample was reverse-transcribed to cDNA with TaqMan^®^ Reverse Transcription Reagents kit (Applied Biosystems, cat# N808-0234, USA) and then 500 ng of cDNA was analyzed using the Maxima SYBR Green qPCR Master Mix kit (Thermo Fisher Scientific Inc., cat# K0252, USA) with an ABI 7500 real-time PCR instrument (Applied Biosystems) according to the manufacturer's instruction. The primers were synthetized by Integrated DNA Technologies (IDT, Belgium) and were as follow: eNOS Fwd (5′-TGT TCG ATG CCC GGG ACT GC-3′) and eNOS Rev (5′-AGC GAA GGT TGC CCC GGT TG-3′); MMP-2 Fwd (5′-CAA AAC GGA CAA AGA GTT GGC A-3′) and MMP-2 Rev (5′-GGT CAA GAT CAC CTG TCT GGG -3′); GAPDH Fwd (5′-CGC TTC GCT CTC TGC TCC TCC-3′) and GAPDH Rev (5′-TGG TGA CCA GGC GCC CAA TAC-3′). PCR was performed at 95°C for 15 s and 60°C for 60 s for 40 cycles. GAPDH was used as an internal standard to normalize mRNA levels for differences in sample concentration and loading. Fold-changes in the expression of each target mRNA relative to GAPDH were calculated based on the threshold cycle (Ct) as 2−Δ(ΔCt), where ΔCt = Ct target − Ct GAPDH and Δ(ΔCt) = ΔCtAdh-EC − ΔCtEC. The post-amplification melting curve analysis was performed to confirm that nonspecific amplification was generated from primer dimers. Quantitative PCR reactions were performed in triplicate and repeated three times.

### Western blotting

Endothelial cells untreated or treated with 2 mM L-NAME (Cayman, cat# 80210) for 1 h were lysed in RIPA buffer [150 mM NaCl, 0.1% Triton X-100, 0.5% sodium deoxycholate, 0.1% SDS (sodium dodecyl sulphate), 50 mM Tris-HCl, pH 8, and Halt protease and phosphatase inhibitor cocktail (Thermo Fisher Scientific Inc., cat# 78443)] for 2 h at 4°C. The homogenates were spun at 24,000 *g* for 15 min at 4°C and the supernatant was collected. Proteins were quantified using the BCA Protein Assay kit (Thermo Fisher Scientific Inc., cat# 23225). Equal amounts of protein extracts (40 μg) were subjected to 10% SDS-PAGE and blotted onto a PVDF membrane (Thermo Fisher Scientific Inc., cat# 88518). After blocking, the membrane was incubated with the following primary antibodies: rabbit anti-phospho-eNOS/NOS3 (Thr495) 1:2000 (Millipore, cat# 04-811, RRID:AB_11211432, USA); rabbit anti-phospho eNOS (Ser1177) 1:1000 (Millipore, cat# 07-428-I, RRID:AB_310608); rabbit anti-collagen IV 1:500 (Abcam, cat# ab6586, RRID:AB_305584, UK); rabbit anti-MMP-2 (Cell Signaling, cat# 4022, RRID:AB_2266622, USA) and rabbit-anti-fibronectin (1:500) (Abcam, cat# ab2413, RRID:AB_2262874); rabbit-anti-ATF3 (1:500) (Abcam, cat# ab87213, RRID:AB_1951498); mouse anti-von Willebrand factor (1:100) (Santa Cruz, cat# sc-365712, USA); and mouse anti-β-actin (1:5000) (Cell Signaling, cat# 3700, RRID:AB_2242334) diluted in blocking buffer overnight at 4°C and followed by incubation with secondary antibodies (1:10,000) (Sigma-Aldrich, cat# A4416, RRID:AB_258167 or cat# A6154, RRID:AB_258284) for 1 h at room temperature. The samples were detected by enhanced chemiluminescence using the SuperSignal West Pico Chemiluminescent Substrate (Thermo Fisher Scientific Inc., cat# 34080). An Alliance mini photodocumentation system from UVITEC (UK) was used to scan the films and UVIBAND MAX v1503b software (UVITEC) was used to measure the amount of protein detected by each antibody. The experiments were performed in duplicate and repeated twice.

### Viability assay (MTT)

For cell viability assays, cells were cultured in 96-well plates (5×10^3^ cells/ well) for 24 h. The cells were treated with L-NAME (2 mM) for 1 h. At the end of the treatment, 100 μL MTT (Sigma-Aldrich, cat# M2003) diluted in F-12 (5 mg/mL) was added to each well, and the cells were incubated for 3 h. The purple-blue MTT formazan precipitate was dissolved in 100 μL DMSO, and absorbance was measured at 540 nm using a microplate reader EZ Read 400 (Biochrom). Experiments were performed in triplicate and repeated twice.

### Adhesion assays

Ninety-six-well tissue culture plates (Corning/Costar, USA) were submitted to the substrate for cell attachment (5, 10, 20, or 30 ug/mL of fibronectin, collagen IV, or laminin) for 2 h at 37°C. The plates were washed with PBS and blocked with 1% BSA in PBS for 1 h at 37°C. Endothelial cells (5×10^4^ in F-12 serum-free medium) untreated or treated with L-NAME (2 mM) were added to the plates and submitted to the substrate for cell attachment for 3 h at 37°C. At the end of incubation, the unattached cells were removed by washing the plates with PBS. Attached cells were fixed in methanol for 20 min and stained with 0.8% Crystal violet (Sigma-Aldrich, cat# c3886) dissolved in 20% ethanol and washed fifteen times with PBS. The dye was then eluted with 50% ethanol 0.1 M sodium citrate, pH 4.2 and measured for absorbance at 540 nm using a microplate reader EZ Read 400 (Biochrom). For control, the non-adhesive substrate was prepared by coating the wells with 1% BSA for 60 min at 37°C. The experiments were performed in triplicate for each dose and repeated three times on different days.

### Invasion assay

The invasion assays were performed on 24-well plates using polycarbonate transwell of 8-um pore size (Greiner Bio-One, cat# 662638, Germany) coated with ECL cell attachment matrix (entactin, collagen IV, and laminin; 1 mg/mL) (Merck Millipore, cat# 08-110). Briefly, the coated inserts were hydrated with warm F-12 without serum in 2.5% CO_2_ at 37°C for 2 h. A total of 5×10^4^ endothelial cells untreated or treated with L-NAME (2 mM) were seeded into the upper chamber containing F-12 medium without serum, and the lower chamber contained F-12 with 10% FCS. The cells were incubated at 37°C in 2.5% CO_2_ for 18 h. Non-invading cells were removed from the upper membrane by scrubbing with a cotton swab. Invading cells were fixed with 4% formaldehyde at room temperature for 5 min and methanol (p.a. 99.8%) for 20 min, stained with DAPI (1:10000) dissolved in PBS for 20 min in darkness, and visualized under an inverted microscope Axio Observer Z1 (Zeiss, Germany). The total number of nuclei stained with DAPI were counted, and the ImageJ software (NIH, USA) was used for quantification and analysis of images.

### Migration assay

Cell migration was evaluated by the wound healing experiment. The assay was performed on 24-well plates. Briefly, a total of 2.5×10^4^ cells were seeded onto each well. After 24 h, ECs in monolayer untreated or treated with L-NAME (2 mM) were wounded with a 200-uL tip and rinsed twice with PBS. Medium was replaced with new serum-free medium. Endothelial migration was observed and photographed at 0 and 4 h after the injury using an inverted microscope (Axio Observer Z1, Zeiss). ImageJ software was used for area quantification. The width of wounds was measured, and relative migration of cells was calculated based on the healing distance. All experiments were performed in triplicate and repeated twice.

### Immunocytochemistry

Cells were cultured on 13.0-mm diameter glass coverslips (5×10^3^ cells/coverslip) in 24-well plates for 2 days followed by treatment with L-NAME (2 mM) for 1 h and fixation with 4% formaldehyde in PBS (pH 7.4) for 20 min at room temperature. The cells were washed four times with PBS and once with PBS containing 0.1 M glycine. They were then permeabilized with PBS containing 1% BSA and 0.01% saponin for 15 min at room temperature. After this step, the cells were washed with PBS and incubated with rabbit anti-fibronectin (1:200) and rabbit anti-collagen IV (1:200) diluted in PBS for 1 h at room temperature. After washing, the cells were incubated with goat anti-rabbit labeled with Alexa Fluor 633 (1:300) (Thermo Fisher Scientific Inc., cat# A21071) and DAPI (2 mg/mL) in PBS containing 0.01% saponin for 20 min at room temperature. The cells were washed, mounted in Fluoromount-G (Southern Biotech, cat# 0100-01, USA), and examined using an inverted confocal laser-scanning microscope (Leica TCS SP8, Leica Microsystems, Germany). The figures in the Results section are the best image of two experiments.

### MMP-2 activity assay

MMP-2 activity was measured with the SensoLyte 520 MMP-2 Assay kit (AnaSpec, cat# 71151, USA) according to the manufacturer's instructions. The supernatants of treated and untreated cells were collected and then incubated with 4-aminophenylmercuric acetate (AMPA) and MMP-2 substrate. The fluorescence intensity at Ex/Em wavelengths of 490/520 nm was used as a measure of MMP-2 activity. Each experiment was repeated twice in triplicates.

### Statistical analysis

All data are reported as means±SE. Statistical significance was assessed using ANOVA. P-values <0.05 were considered statistically significant.

## Results

### Endothelial cells resistant to anoikis overexpressed eNOS and displayed an increase in NO release

**Figure 1:**
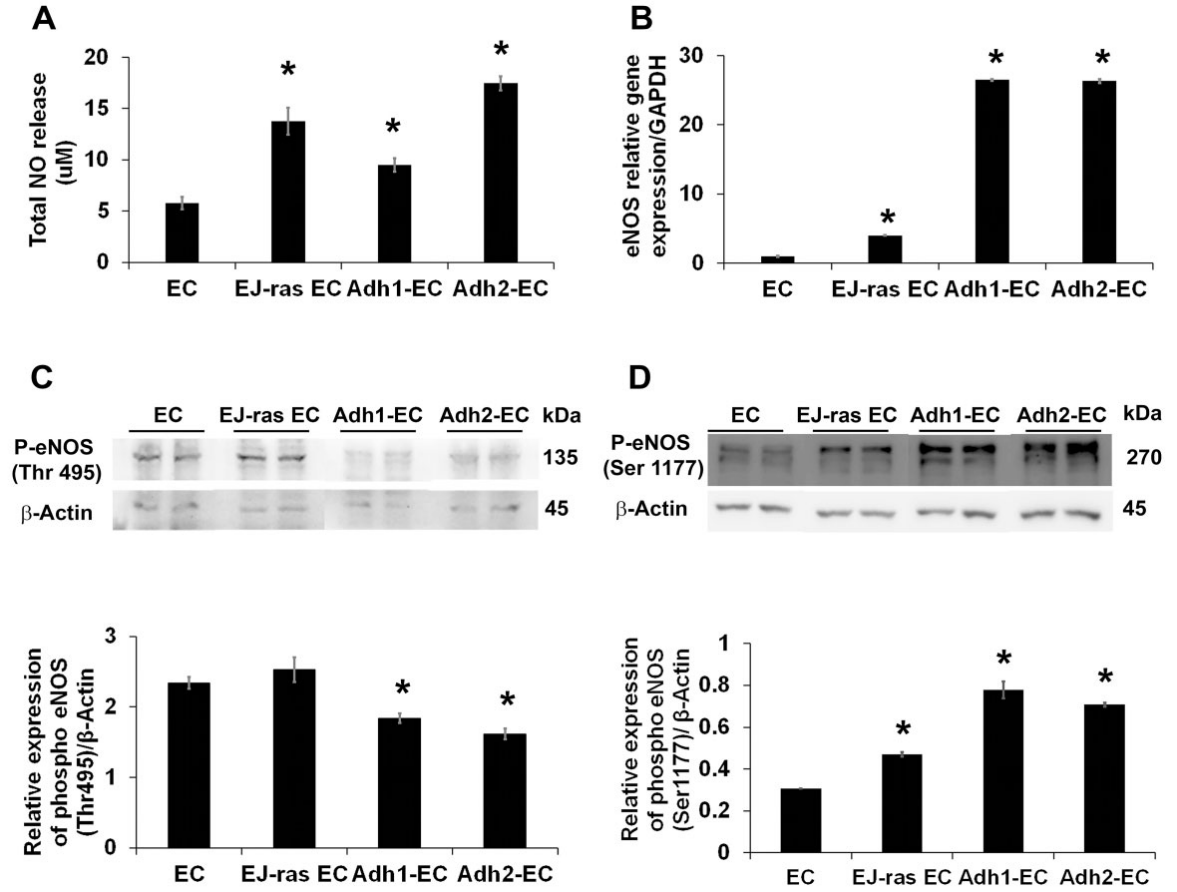
Nitric oxide (NO) release and endothelial nitric oxide synthase (eNOS) expression in EC-derived cell lines. **A**, NO release by EC, EJ-ras EC, Adh1-EC, and Adh2-EC cells. Data were evaluated by NO-analyzer chemiluminescent method. Experiments were performed in duplicate. **B**, Gene expression of eNOS detected by qPCR. GAPDH was used as a loading control. **C**, Protein expression of monomeric eNOS (135 kDa) phosphorylated at threonine 495 (Thr495). **D**, Protein expression of dimeric eNOS (270 kDa) phosphorylated at serine 1177 (Ser1177). The experiments were performed in duplicate and repeated twice. EC: parental endothelial cells; EJ-ras EC: EJ-ras transfected endothelial cells; Adh1-EC and Adh2-EC: anoikis-resistant endothelial cells. Data are reported as means±SE. *P<0.05 compared to EC (ANOVA).

Figure 1A shows an increase in the NO production by anoikis-resistant cell lines (Adh1-EC and Adh2-EC) and tumorigenic cell line (EJ-ras EC) compared to EC.

The results of PCR analysis presented in Figure 1B revealed that the acquisition of anoikis resistance increases the *eNOS* gene expression in endothelial cells. Anoikis-resistant endothelial cells showed a significant decrease in the inactive monomeric eNOS protein phosphorylated at Threonine 495 (Thr495) (Figure 1C) compared to EC. This was accompanied by a significant increase in the levels of active dimeric eNOS phosphorylated at Serine 1177 (Ser1177) compared to EC (Figure 1D). Similar results were observed in tumorigenic EJ-ras EC cells.

Also, to investigate the occurrence of endothelial-to-mesenchymal transition (EndMT) in anoikis-resistant endothelial cells, the protein expression of the von Willebrand factor was evaluated, and the results are presented as Supplementary Figure S1.

### L-NAME inhibited NO production without altering cell viability in anoikis-resistant endothelial cells

L-NAME treatment did not affect the cell viability of the cell lines (Supplementary Figure S2A) and effectively reduced NO production levels around 50% in all cell lines (Supplementary Figure S2B). Based on these results, L-NAME was used in all following experiments to investigate the consequences of inhibition of NO production in various cellular functions.

### L-NAME treatment affected adhesiveness, invasiveness, and migration in anoikis-resistant endothelial cells

**Figure 2:**
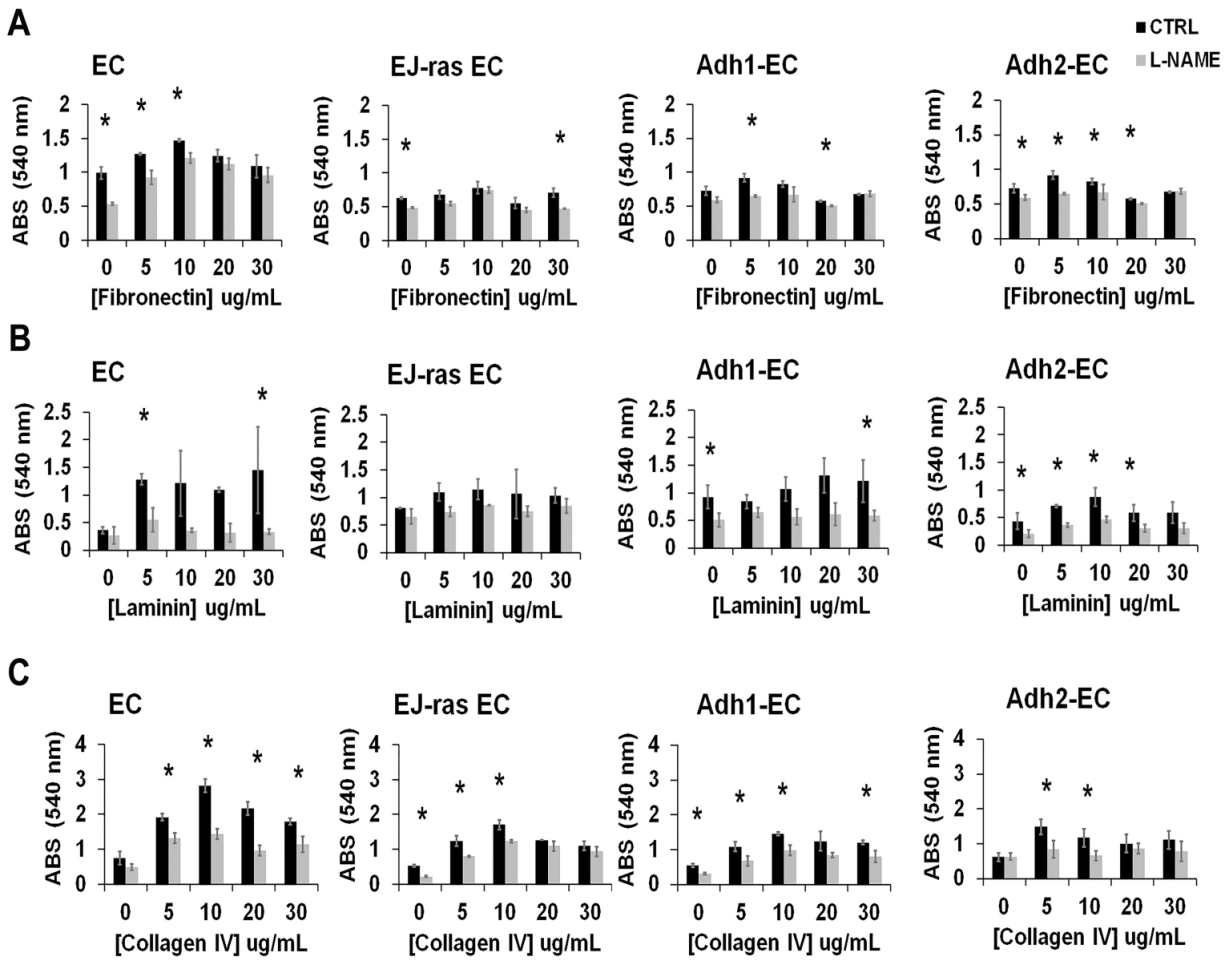
L-NAME treatment decreased cell adhesiveness to fibronectin, laminin, and collagen IV in EC-derived cell lines. **A**, **B**, and **C**, Adhesion assays of parental (EC), EJ-ras transfected endothelial cells (EJ-ras EC), and anoikis-resistant endothelial cells (Adh1-EC and Adh2-EC) on fibronectin, laminin, and collagen IV, respectively, after L-NAME treatment (2 mM for 1 h). The experiments were performed in triplicate for each dose and repeated three times on different days. ABS: absorbance; CTRL: Control; EC: parental endothelial cells. Data are reported as means±SE. *P<0.05 compared to control (*t*-test).

**Figure 3:**
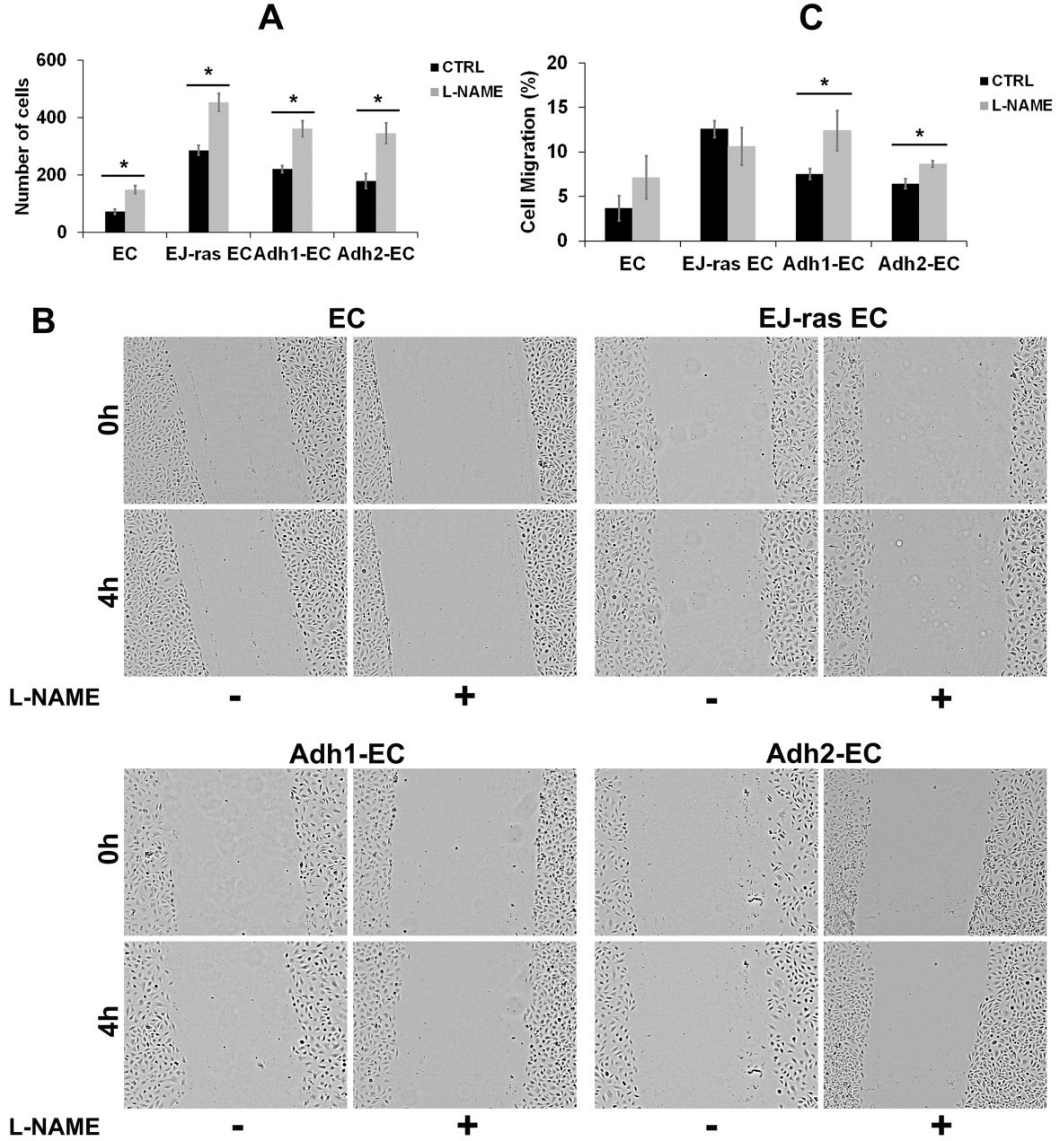
L-NAME treatment increased invasiveness and cell migration in EC-derived cell lines. **A**, Invasiveness after nitric oxide (NO) release inhibition in EC, EJ-ras EC, Adh1-EC, and Adh2-EC cells. **B**, Images of wound healing experiment 0 and 4h after the injury of untreated and L-NAME-treated EC-derived cell lines. **C**, Relative cell migration of EC, EJ-ras EC, Adh1-EC, and Adh-2 EC cells after L-NAME treatment. Data were normalized by time 0 h (0%). All experiments were performed in triplicate and repeated twice. CTRL: Control; EC: parental endothelial cells; EJ-ras EC: EJ-ras transfected endothelial cells; Adh1-EC and Adh2-EC: anoikis-resistant endothelial cells. Data are reported as means±SE. *P<0.05 compared to control (*t*-test).

The L-NAME treatment and consequent inhibition of NO release led to a decrease of the adhesiveness to fibronectin (Figure 2A), laminin (Figure 2B), and collagen IV (Figure 2C) in anoikis-resistant endothelial cells and EJ-ras EC. Loss of adhesiveness is directly associated with high invasiveness and high migratory potential. As shown in Figure 3A, invasiveness increased in all cell lines after treatment with L-NAME.

Similar results were found in migratory analysis. Figure 3B shows images of cell migration after injury in treated and untreated cells. Figure 3C shows a graphic analysis of cell migration, and it is possible to observe an increase in cell migration after L-NAME treatment in Adh1-EC and Adh2-EC cells.

### L-NAME treatment affected fibronectin and collagen IV protein expression

**Figure 4:**
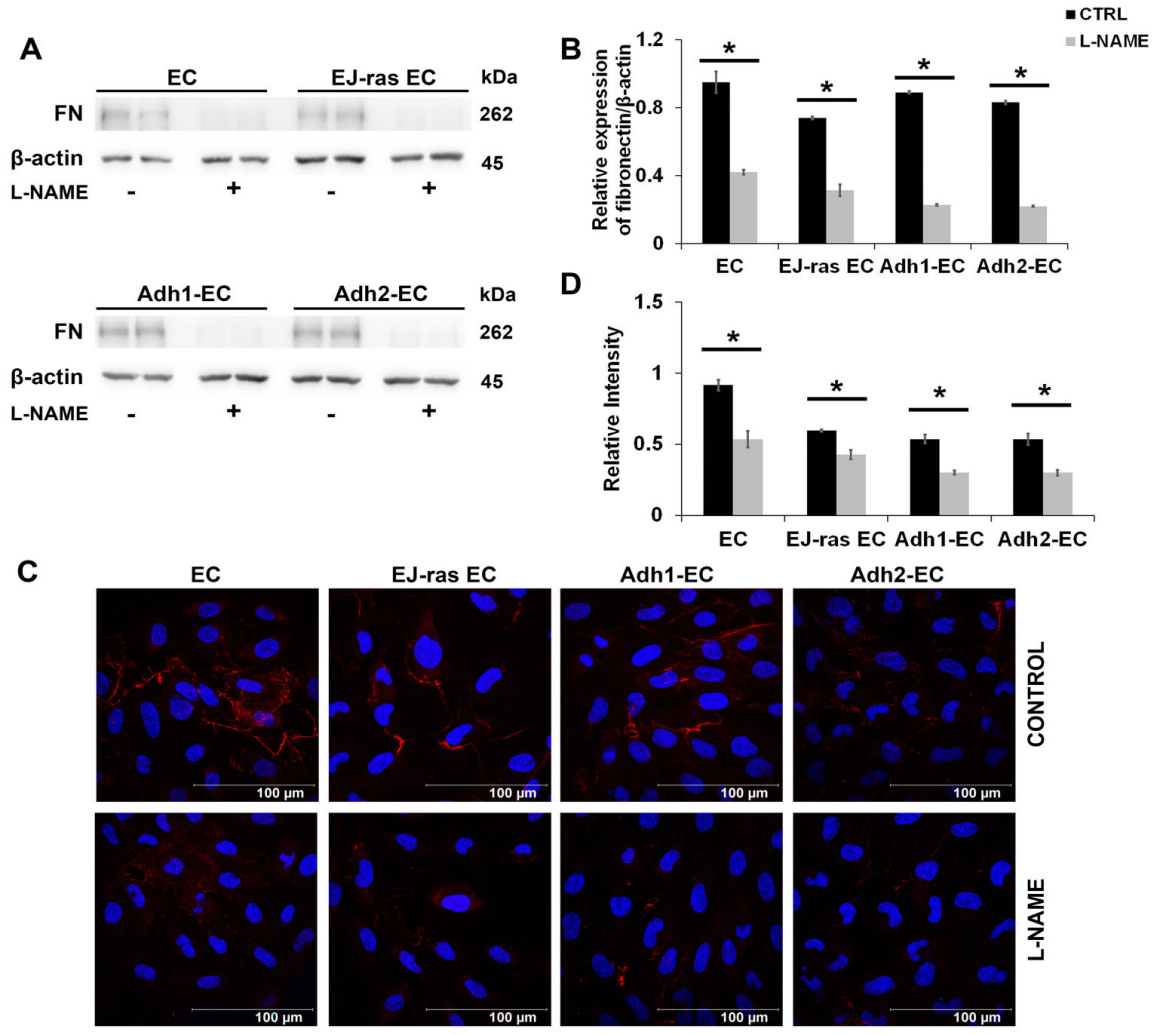
L-NAME treatment decreased fibronectin expression in EC-derived cell lines. **A**, Western blot images of fibronectin expression after NO release inhibition in EC, EJ-ras EC, Adh1-EC, and Adh2-EC cells. **B**, Histogram depicting fibronectin protein levels normalized to β-actin. The experiments were performed in duplicate and repeated twice. **C**, Immunofluorescent analysis of fibronectin by immunocytochemistry of EC, EJ-ras EC, Adh1-EC, and Adh-2 EC cells after L-NAME treatment (scale bar 100 μm). **D**, Histogram depicting fibronectin protein levels normalized by DAPI fluorescence. Fibronectin was stained with anti-fibronectin antibody followed by secondary Alexa Fluor 633-labelled antibody (red). Nucleus is stained with DAPI (blue). Each figure shows the best image of two experiments. CTRL: Control; EC: parental endothelial cells; EJ-ras EC: EJ-ras transfected endothelial cells; Adh1-EC and Adh2-EC: anoikis-resistant endothelial cells. Data are reported as means±SE. *P<0.05 compared to control (*t*-test).

Alterations in invasiveness and adhesiveness could be related to extracellular matrix degradation and remodeling. To confirm this hypothesis, we performed western blot and immunofluorescence analyses to evaluate location and protein expression of important endothelial matrix molecules as fibronectin and collagen IV after inhibition of NO release. As shown in Figure 4A and B, L-NAME treatment of endothelial cells drastically reduced fibronectin protein levels. As expected, immunocytochemistry analyses showed that fibronectin (red) was localized in the extracellular matrix and its expression decreased significantly in L-NAME-treated cells (around 41.5% in EC, 28.5% in EJ-ras EC, 44.0% in Adh1-EC, and 43.8 in Adh2-EC) (Figure 4C and D).

**Figure 5:**
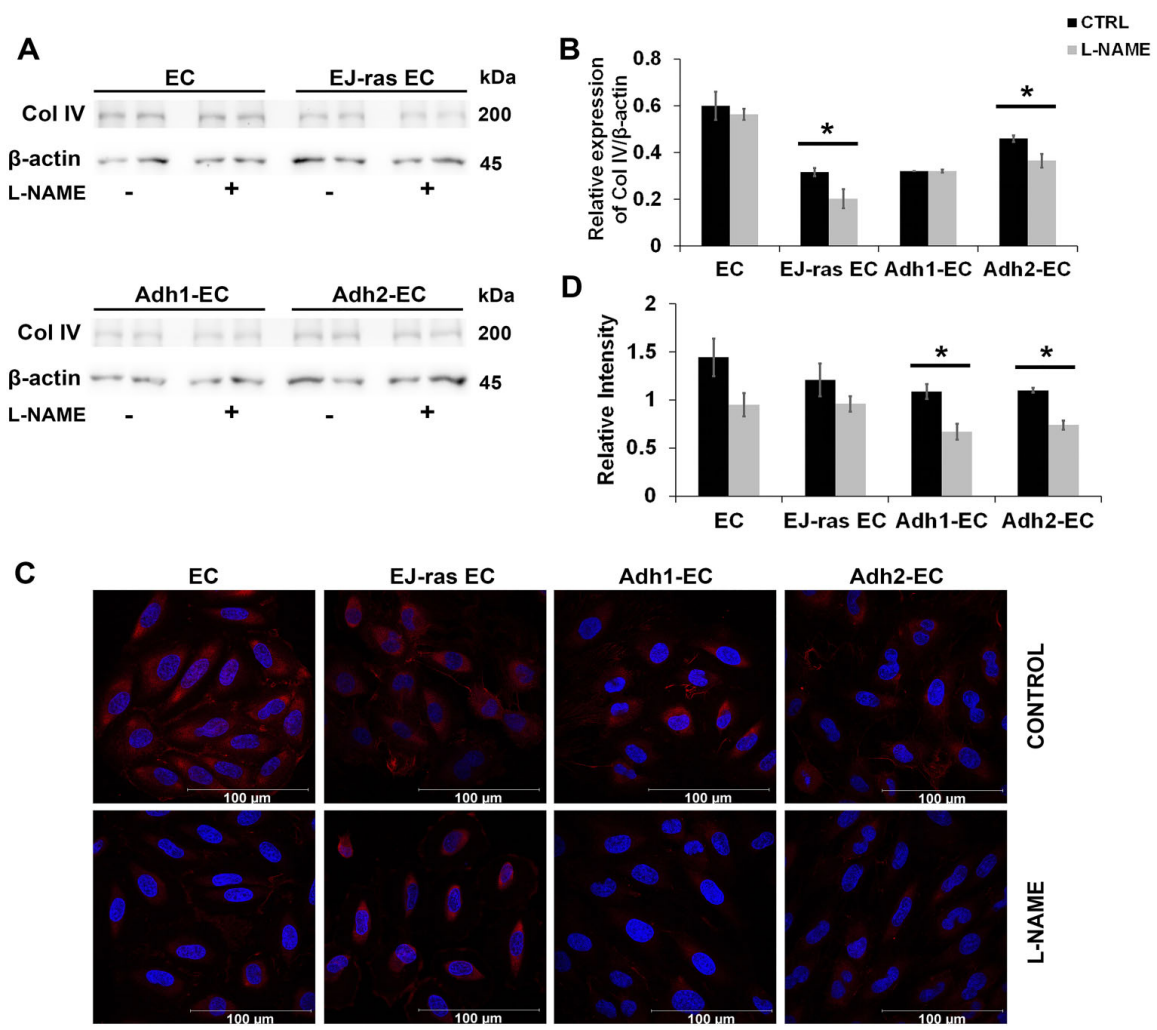
L-NAME treatment decreased collagen IV expression in EC-derived cell lines. **A**, Western blot images of collagen IV expression after nitric oxide (NO) release inhibition in EC, EJ-ras EC, Adh1-EC, and Adh2-EC cells. **B**, Histogram depicting collagen IV protein levels normalized to β-actin. **C**, Immunofluorescent analysis of collagen IV by immunocytochemistry of EC, EJ-ras EC, Adh1-EC, and Adh-2 EC cells after L-NAME treatment (scale bar 100 μm). **D**, Histogram depicting collagen IV protein levels normalized by DAPI fluorescence. Collagen IV was stained with anti-collagen IV antibody followed by secondary Alexa Fluor 633-labelled antibody (red). Nucleus is stained with DAPI (blue). Each figure shows the best image of two experiments. CTRL: Control; EC: parental endothelial cells; EJ-ras EC: EJ-ras transfected endothelial cells; Adh1-EC and Adh2-EC: anoikis-resistant endothelial cells. Data are reported as means±SE. *P<0.05 compared to control (*t*-test).

Inhibition of NO production affected not only fibronectin protein levels but also collagen IV expression (Figure 5A and B), especially in anoikis-resistant endothelial cells. Collagen IV protein levels were greatly reduced after L-NAME treatment (around 34.1% in EC, 20.6% in EJ-ras EC, 38.3% in Adh1-EC, and 32.8 in Adh2-EC), as shown in immunocytochemistry analyses (Figure 5C and D). The results presented here suggested the involvement of NO in endothelial extracellular matrix remodeling.

### L-NAME treatment affected MMP-2 expression and activity in anoikis-resistant endothelial cells

**Figure 6:**
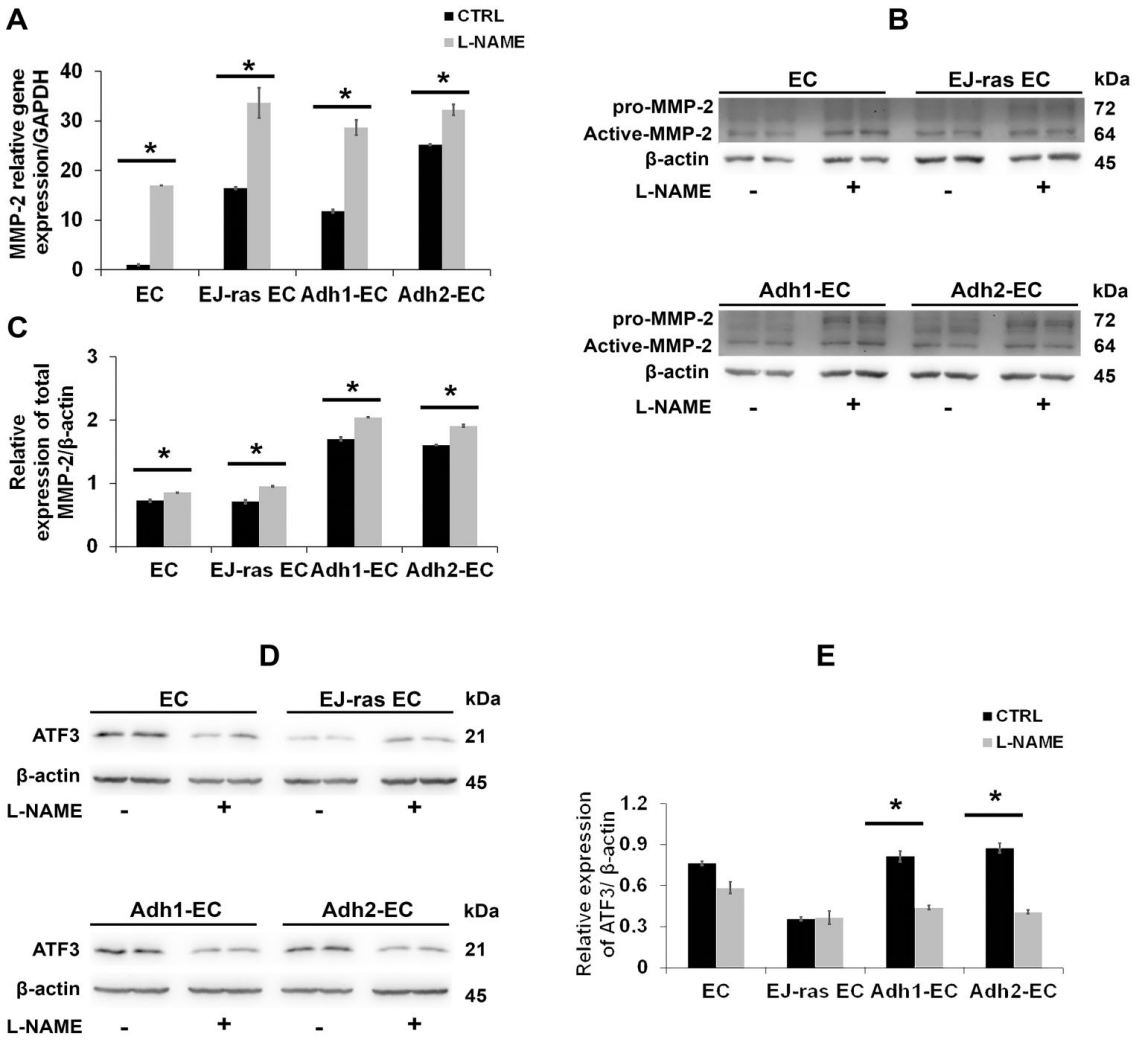
L-NAME treatment up-regulates MMP-2 gene and protein expression, and down-regulates activating transcription factor 3 (ATF3) protein expression in EC-derived cell lines. **A**, *MMP-2* gene expression after nitric oxide (NO) release inhibition of EC, EJ-ras EC, Adh1-EC, and Adh2-EC cells. **B**, Western blot images of MMP-2 expression after NO release inhibition in EC-derived cell lines. **C**, Histogram depicting MMP-2 protein levels normalized to β-actin. **D**, Western blot images of ATF3 expression after NO release inhibition in EC-derived cell lines. **E**, Histogram depicting ATF3 protein levels normalized to β-actin. The experiments were performed in duplicate and repeated twice. CTRL: Control; EC: parental endothelial cells; EJ-ras EC: EJ-ras transfected endothelial cells; Adh1-EC and Adh2-EC: anoikis-resistant endothelial cells. Data are reported as means±SE. *P<0.05 compared to control (*t*-test).

Based on previous results and given the importance of MMP-2 in collagen and fibronectin degradation, we investigated the effect of L-NAME treatment on MMP-2 gene and protein expression. The reduction in NO release positively regulated *MMP-2* gene expression in all cell lines (Figure 6A). Consistent with these findings, western blot analyses demonstrated that protein expression of MMP-2 increased after L-NAME treatment in all cell lines (Figure 6B and C). Furthermore, MMP-2 activity analysis showed an increase in MMP-2 activity around 37% in EC, 11% in EJ-ras EC, 27% in Adh1-EC, and 15% in Adh2-EC after L-NAME treatment (Supplementary Figure S3).

### ATF3 transcription factor was the key in MMP-2 expression regulation by NO in anoikis-resistant endothelial cells

Using western blot analyses, we investigated the correlation between the increase in MMP-2 expression and activating transcription factor 3 (ATF3) expression after L-NAME treatment. Results are presented in Figure 6D and E and show a decrease in ATF3 expression after L-NAME treatment in anoikis-resistant endothelial cells.

## Discussion

Recently, we reported that anoikis-resistant endothelial cells exhibit morphological alterations, poor adhesion to collagen IV, laminin and fibronectin, high proliferation rate and invasiveness, low rate of apoptosis, and cell cycle dysregulation (2). Furthermore, anoikis-resistant cell lines present up-regulated PI3K/Akt and Ras/ERK pathways. This is accompanied by an extensive matrix remodeling (5).

Anoikis occurs after the detachment of anchorage-dependent cells from their adjacent extracellular matrix. It has been proposed that tumor cells develop several strategies to evade this type of apoptosis. Anoikis-resistant cancer cells are able to invade easily and migrate to distant metastatic sites. These cells may undergo metastasis and facilitate tumor formation in other sites (19). The mechanisms underlying anoikis resistance in tumor cells still need further elucidation.

Here, we show that endothelial cell lines resistant to anoikis (Adh1-EC and Adh2-EC) up-regulated eNOS enzyme expression and increased NO release compared to the EC parental cell line. Similar results were found in tumorigenic endothelial cells (EJ-ras EC). Reactive oxygen species (ROS) are mediators of a number of cellular signaling processes related to tumor development. In fact, the chronic generation of NO and ROS maintain the oxidative and nitrosative stress in tumors (20). However, NO has been reported as a molecule with dual effects in cellular proliferation, invasion, apoptosis, migration, angiogenesis, and other important processes in cancer biology. Depending on its levels and the tumor microenvironment, this molecule displays both pro- and anti-tumorigenic roles (21).

Endothelial cells resistant to anoikis up-regulated *eNOS* gene expression, increased eNOS protein expression and activation by dimerization, phosphorylation at Ser1177, and dephosphorylation at Thr495. These results were also very similar to the ones found in transformed endothelial cells (EJ-ras EC). The phosphorylation of eNOS at Ser1177 is crucial to increase enzyme performance. This phosphorylation reduces the enzyme's Ca^2+^- dependence, increases the electron flux from the reductase domain to the oxygenase domain, and consequently increases the NO production (22). In contrast, phosphorylation at Thr495 decreases eNOS activity by increasing Ca^2+^- CaM dependence acting as an inhibitory mechanism (23). Thus, the decrease in the Thr495 phosphorylation levels contributes even more to the high activity of eNOS in these cells. The up-regulation of eNOS expression and activity in anoikis-resistant endothelial cells can be associated with the overexpression of Akt in those cells as previously shown (5). Fulton et al. (24) have demonstrated that the serine/threonine protein kinase Akt (protein kinase B) can directly phosphorylate eNOS on Ser1179 in bovine cells and activate the enzyme.

The role of NO in adhesiveness, invasiveness, and cell migration was investigated in EC-derived cell lines using L-NAME, an L-arginine analogue that acts as nitric oxide synthase inhibitor.

Anoikis-resistant endothelial cells showed a decrease in adhesiveness and an increase in invasiveness and cell migration after inhibition of NO synthesis by L-NAME treatment (2 mM for 1 h). This was accompanied by an increase in MMP-2 gene expression and protein activity. Besides that, L-NAME treatment led to a reduction in fibronectin and collagen IV present in the ECM composition. These results suggested that the increase in MMP-2 gene and protein expression was associated with degradation of fibronectin and collagen IV thus contributing to cell migration by eliminating the surrounding ECM and basement membrane barriers.

ECM degradation is a vital step in metastasis and involves numerous types of proteases. The most important proteases are the MMPs called matrixins (13). Metalloproteinases are able to degrade most components of the ECM and basal membrane, such as collagen, fibronectin, and elastin. As MMPs have thrombospondin motifs, they act as metalloproteinases and disintegrins. If left unchecked, the strong action of these enzymes can have devastating consequences on tissues. Besides being regulated at the transcriptional and translational levels, ECM remodeling enzymes are also post-translationally regulated by their functionally inhibitive prodomains and selective proteinase inhibitors (25).

Adhesion, invasiveness, and cell migration are related to MMP-2 expression and activation, and MMP-2 can be indirectly influenced by NO levels. In fact, major cancer hallmarks including migration, invasiveness, apoptosis, anoikis resistance, and angiogenesis have been shown to be affected by NO levels in the tumor microenvironment. Thus, NO can modulate molecular mechanisms and cross talks during numerous stages of cancer. It has also been shown to affect diverse gene expressions at transcriptional and translational levels (26).

Several studies have confirmed that NO plays an important role in cell adhesion and migration. NO release has been shown to enhance cell migration and decrease cell adhesion in some cell types (27) and conversely to reduce cell migration and increase cell adhesion in other cell types (28). It is worth noting that, in addition to concentration, other factors such as the duration of NO exposure and the kinetic behaviors of NO are also key determinants in NO's biological signaling (29). Our results suggested that the decrease in NO release after L-NAME treatment indirectly affected MMP-2 gene and protein expression.

*MMP-2* promoter contains a conserved consensus p53-binding site previously shown to up-regulate *MMP-2* expression (30). P53 transcription factor recognizes a specific consensus DNA sequence which consists of two copies of the 10-bp motif 5′-PuPuPuC(A/T) (T/A)GPyPyPy-3′, separated by 0 to 13 bp. P53 accurately binds to this sequence and trans-activates expression of the target genes. Thus, P53 binds to its consensus binding site present in the promoter of the gene encoding type IV collagenase (*MMP-2*) and trans-activates this gene expression (30).

Yan et al. (31) have shown that *MMP-2* gene expression is down-regulated by ATF3 via the suppression of p53 trans-activation on the *MMP-2* promoter region. ATF3 is one of a few proteins rapidly induced as part of the cellular response to a wide range of stresses (32). Since ATF3 is known to be stress inducible, it is conceivable that this protein is regulated by factors involved in stress response and inflammation such as nitric oxide; for this reason, we decided to evaluate ATF3 expression as a connection between NO and downregulation of MMP-2. ATF3 is a member of the ATF/CREB transcription factors family. This group of basic region-leucine zipper (bZip) proteins is reported to be involved in several stress responses (33). The ability of ATF3 homo- and heterodimers regulating the expression of several genes occurs due to their capacity to specifically bind to the ATF/CREB and AP-1 motifs. Transcriptional repression of MMP-2 is achieved by attenuating ATF3 with p53 transcriptional activity, but not DNA binding. Thus, p53-dependent trans-activation of MMP-2 is affected (31).

The anoikis-resistant endothelial cells have shown a decrease in ATF3 expression as a consequence of NO production inhibition. The detailed mechanisms of ATF3 induction by NO are not clear. Several studies have demonstrated that alterations in NO production regulates ATF3 expression and consequently interferes in *MMP-2* expression in endothelial cells (17,34), glioblastoma (35), and mouse brain endothelial cells (36).

Alternatively, MMP-2 can be activated via a non-proteolytic process, which involves the S-glutathiolation of a cysteine sulfhydryl moiety in its propeptide portion by peroxynitrite (ONOO-) originated from the prooxidant reaction of NO (16). This occurs in the presence of intracellular glutathione and at low (0.3-1.0 μM) ONOO- concentrations. Conversely, high concentrations of ONOO- (>100 μM) can lead to a severe and irreversible protein oxidation of cysteine and other residues causing the loss of MMP-2 catalytic activity (16,37).

The combined results provided evidence that inhibition of NO release in endothelial cells resistant to anoikis after L-NAME treatment increased invasiveness, migration, and fibronectin and collagen IV degradation via modulation of MMP-2 expression. It is hypothesized that MMP-2 expression was regulated by ATF3 interaction with the p53 protein, a trans-activator of *MMP-2* gene expression. This mechanism was very well described by Yan et al. (31). Furthermore, Chen and Wang found strong evidence that NO acts as a regulator of migration in endothelial cells. They have shown that NO inhibits MMP-2 expression via the induction of ATF3 in endothelial cells (17).

Numerous investigations have shown that low levels of NO promote tumor growth by stimulating angiogenesis and promoting metastasis, improving cancer cell progression, while higher concentrations of NO depress cancer progression by inducing apoptosis, arresting the metastatic and angiogenic cascades, and sensitizing tumors to chemo-, radio-, or immunotherapy (29). Smeda et al. (38) connected NO levels with the EndMT process. The expression of the von Willebrand factor (vWF), a glycoprotein recognized as a marker for endothelial cells, was evaluated in order to confirm the endothelial phenotype of the cells used in this model and discard the evidence of EndMT in Adh-EC cells (Supplementary Figure 3).

The anoikis-resistant phenotype in endothelial cells is supported by several alterations in the molecular machinery, which include differential expression and activation of integrins, proteoglycans, proteases, signaling pathways, and MEC proteins (2). Our results suggested that the increase in NO release by anoikis-resistant endothelial cells acts as a protective response to restrict MMP-2 activation, even though this is not enough to repress its action in invasiveness promotion. However, without the increase in NO release by anoikis-resistant endothelial cells, the invasiveness and migration potential would be even higher than found after L-NAME treatment.

In conclusion, this study revealed that the acquisition of anoikis resistance by endothelial cells induced eNOS up-regulation. NO might play a role in the modulation of the anoikis process and in the regulation of anoikis resistance due to its effect on cell adhesion, migration, invasion, and ECM degradation. Therefore, a better understanding of the ideal concentration of NO, which may promote or arrest anoikis resistance, may provide a novel therapeutic strategy for cancer therapy and prevention of metastasis. Alone or in combination with other antineoplastic compounds, NO can be used as an alternative to conventional treatments.

## Supplementary Material

Click here to view [pdf].
